# Dendritic Epidermal T Cells in Allergic Contact Dermatitis

**DOI:** 10.3389/fimmu.2020.00874

**Published:** 2020-05-19

**Authors:** Veronika Mraz, Carsten Geisler, Charlotte Menné Bonefeld

**Affiliations:** The LEO Foundation Skin Immunology Research Center, Department of Immunology and Microbiology, Faculty of Health and Medical Sciences, University of Copenhagen, Copenhagen, Denmark

**Keywords:** allergic contact dermatitis, skin, inflammatory disease, contact allergens, dendritic epidermal T cells, γδ T cells, stress proteins

## Abstract

Allergic contact dermatitis (ACD) is a common inflammatory skin disease with a prevalence of approximately 20% in the European population. ACD is caused by contact allergens that are reactive chemicals able to modify non-immunogenic self-proteins to become immunogenic proteins. The most frequent contact allergens are metals, fragrances, and preservatives. ACD clinically manifests as pruritic eczematous lesions, erythema, local papules, and oedema. ACD is a T cell-mediated disease, involving both CD4^+^ and CD8^+^ T cells. In addition, γδ T cells appear to play an important role in the immune response to contact allergens. However, it is debated whether γδ T cells act in a pro- or anti-inflammatory manner. A special subset of γδ T cells, named dendritic epidermal T cells (DETC), is found in the epidermis of mice and it plays an important role in immunosurveillance of the skin. DETC are essential in sensing the contact allergen-induced stressed environment. Thus, allergen-induced activation of DETC is partly mediated by numerous allergen-induced stress proteins expressed on the keratinocytes (KC). Several stress proteins, like mouse UL-16-binding protein-like transcript 1 (Mult-1), histocompatibility 60 (H60) and retinoic acid early inducible-1 (Rae-1) α-ε family in mice and major histocompatibility complex (MHC) class I—chain-related A (MICA) in humans, are upregulated on allergen-exposed KC. Allergen-induced stress proteins expressed on the KC are consequently recognized by NKG2D receptor on DETC. This review focuses on the role of γδ T cells in ACD, with DETC in the spotlight, and on the role of stress proteins in contact allergen-induced activation of DETC.

## Allergic Contact Dermatitis

Allergic contact dermatitis (ACD), an inflammatory dermatosis caused by contact of the skin with substances from the environment, is known to humankind since ancient times (1). One of the first cases come from the first century A.D., where patients experienced pruritic eczema upon cutting pine trees (2).

ACD is a type IV hypersensitivity mainly orchestrated by allergen-specific T cells (3), and it is one of the most frequent forms of inflammatory skin diseases. ACD clinically manifests as pruritic eczematous lesions, erythema, local papules and oedema (4–6). In most cases, the dermatitis is localized to the site of contact with the contact allergen; however, systemic reactions can also occur (7, 8). In chronic lesions, the skin is scaly and thicker with erythema and often vesicles (9).

Allergy to contact allergens is diagnosed by patch testing. The most recent systematic review and meta-analysis conducted by *Alinaghi et al*. states that 20.1% of patch tested individuals from the general population suffer from contact allergy with twice as high prevalence in women than in men (10). The most common contact allergens are nickel, cobalt, fragrance allergens, chromium, *p-*phenylenediamine, methylchloroisothiazolinone/methylisothializanone, and colophonium (10). Treatment of ACD is still only symptomatic often including anti-inflammatory corticosteroids (11). A deeper understanding of the immune cells and signaling pathways involved in the response to contact allergens is central for the development of more specific treatments for ACD.

## Immune Response to Contact Allergens

**Figure 1:**
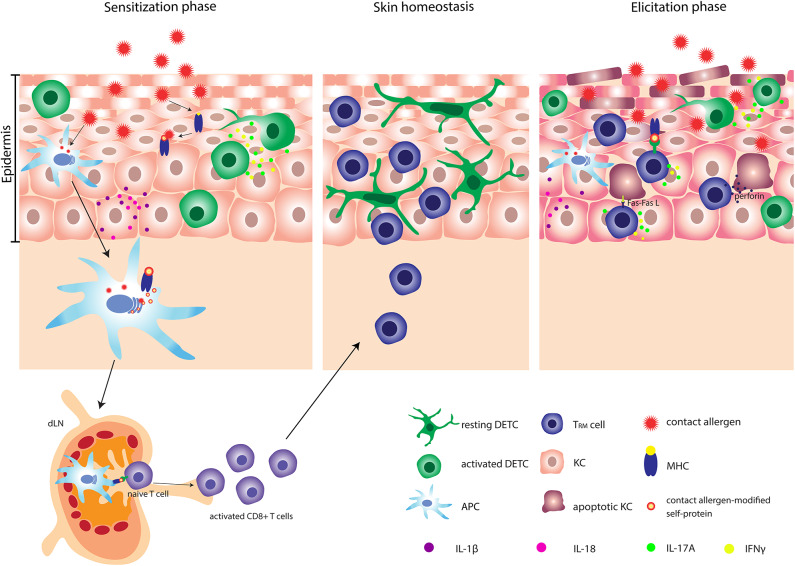
Pathophysiology of allergic contact dermatitis (ACD). After penetrating the skin, contact allergens react with or modify self-proteins into immunogenic proteins. Contact allergen-stressed KC produce profound amount of IL-1β and IL-18 and activated DETC produce IL-17A and IFN-γ. Simultaneously, APC are activated and can subsequently carry contact allergen-modified self-protein-MHC complexes to the dLN, where the priming of naïve T cells occur. Activated CD8^+^ T cells undergo clonal expansion and several memory subsets are developed. One specific subset of memory CD8^+^ T cells e.g., T_RM_ cells migrates back to the site of primary allergen exposure. At the second exposure to the contact allergen, a faster and stronger inflammatory response is induced. Activated DETC and T_RM_ cells produce significant amounts of IL-17A and IFN-γ and stressed KC produce large amounts of IL-1β. Additionally, T_RM_ cells can induce apoptosis in allergen-modified KC via either the Fas-FasL-dependent or the perforin-dependent pathway. DETC, dendritic epidermal γδ T cell; APC, antigen presenting cell; T_RM_ cells, tissue-resident memory T cells; KC, keratinocyte.

Much of our knowledge on the immune response to contact allergens comes from studies using mouse models. Mouse models of ACD are often described as contact hypersensitivity (CHS) models. As ACD is a result of CHS, the abbreviations ACD and CHS will be used interchangeably in this review. ACD is mainly driven by T cells and its pathophysiology is divided into two phases, namely the sensitization and the elicitation/challenge phase (Figure 1). After penetrating the skin, contact allergens modify self-proteins into immunogenic proteins. The stability of the immunogenic proteins is crucial for a proper induction of an acquired immune response (12). Skin inflammation is rapidly induced upon exposure of the skin to contact allergens with interleukin (IL) IL-1β and IL-18 being essential in the response (13–17). Blocking IL-1β with neutralizing antibodies prior to sensitization with TNCB resulted in decreased ear swelling after challenge to TNCB, indicating that IL-1β plays an important role in the induction of ACD in mice (18). IL-1β-deficient mice showed no footpad swelling following sensitization with low concentration of TNCB and required high concentrations of TNCB to develop significant footpad swelling (19). Treatment of mice with the IL-1 receptor (IL-1R) antagonist anakinra suppressed the ear swelling in response to DNFB (20), and a similar effect was seen in IL-1R-deficient mice in response to TNCB (15). In brief, IL-1β clearly seems to be an important mediator in both the sensitization and elicitation phases (15, 18–20).

The inflammatory response leads to activation and migration of antigen-presenting cells (APC) from the skin via afferent lymphatic vessels to the draining lymph nodes (dLN), where the priming and activation of naïve T cells occur (21–23). Following activation, T cells proliferate and differentiate into effector and memory T cells. By the generation of memory T cells an individual has become sensitized to the allergen and subsequent exposures to the same allergen will induce a challenge/elicitation response.

Both CD4^+^ and CD8^+^ T cells are important mediators of the immune response to contact allergens. However, whereas CD8^+^ T cells are involved in pro-inflammatory responses, CD4^+^ T cells can mediate both pro- and anti-inflammatory responses (24, 25). CD4^+^ and CD8^+^ T cells mediate inflammation via production of IFNγ and IL-17A (25–31). Different subsets of memory T cells, including circulating central and effector memory T cells and tissue-resident memory T (T_RM_) cells develop during the sensitization phase (32). A specific subset of CD8^+^ T_RM_ cells is generated locally in the epidermis following exposure of the skin to contact allergens (32–34). Interestingly, a faster and stronger inflammatory response is induced in allergen-experienced skin compared to allergen-unexperienced skin. The increased response correlates with the production of IFNγ and IL-17A by T_RM_ cells (33).

## Role of γδ T Cells in ACD

**Figure 2:**
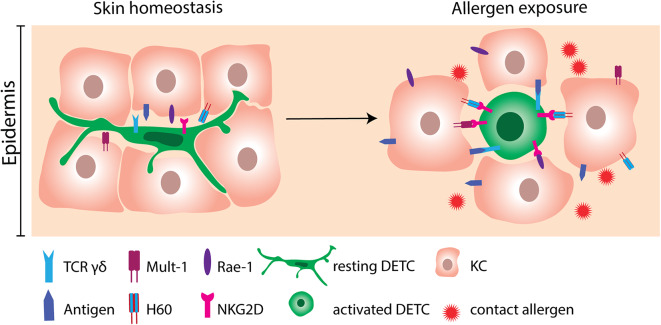
Activation of DETC by contact allergen-induced stress molecules. DETC constantly monitor the epidermis with their dendrites in order to respond rapidly to the incoming environmental triggers. At homeostatic conditions, expression of stress molecules on KC is scarce. Following allergen exposure, KC greatly upregulate NKG2D ligands as Mult-1, H60 and Rae-1. NKG2D ligands consequently bind to NKG2D receptors expressed on DETC. Engagement of NKG2D is not sufficient to activate DETC. Therefore, interaction of the γδ TCR with yet unknown antigens probably needs to occur simultaneously. DETC, dendritic epidermal γδ T cell; KC, keratinocyte.

γδ T cells are unconventional T cells (35, 36) and represent only a minor fraction of the T cells in the blood. However, γδ T cells populate non-lymphoid tissues, like the skin at a high frequency (37–42). A specific subset of γδ T cells is found in mouse epidermis, namely the dendritic epidermal T cells (DETC) that play an important role in skin homeostasis and repair (42–44). The DETC have a highly restricted TCR repertoire, expressing the invariant Vγ3Vδ1 TCR (35, 36, 45). An alternative nomenclature for γδ TCR exists in which DETC are Vγ5Vδ1 (46). Thus, DETC are able to recognize only a limited pool of antigens (35, 36, 47). The antigens recognized by the DETC are yet to be discovered (42, 44, 48–52). However, DETC possess other receptors that recognize a variety of stress-induced molecules and heat shock proteins in a MHC-independent manner (53, 54). This characteristic makes DETC efficient responders to various environmental triggers (Figure 2).

The role of γδ T cells, including DETC, in ACD is debated (please see Table 1 for an overview). Some studies have suggested that γδ T cells play a pro-inflammatory role (20, 55–59, 67), whereas other studies have suggested an anti-inflammatory role of γδ T cells in ACD (63–66). A role of γδ T cells in the response to contact allergens was first shown by adoptive transfer of T cells isolated from the dLN of sensitized mice into allergen-naïve mice (55–57). It was shown that γδ T cells assist αβ T cells in the transfer of CHS from sensitized to naïve mice (55–57), as adoptive transfer of only αβ T cells was not sufficient to transfer CHS. Both αβ and γδ T cells were required to transfer CHS (55, 57). It can be speculated, that the γδ and αβ T cells rely on each other with the production of different cytokines and chemokines required for the migration to allergen-exposed sites. Interestingly, transfer of αβ T cells from mice sensitized with TNP-Cl together with γδ T cells from mice sensitized with oxazolone (OXA) to allergen-naive mice, which were subsequently challenged with TNP-Cl, resulted in increased CHS, indicating that γδ T cells assist αβ T cells in an allergen-unspecific manner (55).

**Table 1:** Pro-inflammatory and anti-inflammatory roles of γδ T cells. DETC; dendritic epidermal γδ T cell.

**Species**	**Model**	**Cell type**	**Cell markers**	**Localization**	**Effect**	**Reference**
**Pro-inflammatory role in ACD**						
Mouse	*In vivo* depletion with anti-TCRδ (UC7-13D5) in CBA/J mice	γδ T cells	TCR γδ (UC7-13D5)	Skin	Assist αβ T cells in the transfer of CHS in an allergen-unspecific manner	(55)
	Adoptive transfer of allergen-derivatized lymphocytes in CHS model in CBA/J mice		TCR γδ (GL3)			(56)
	*In vivo* allergen-derivatized γδ T cells for adoptive transfer to CBA/J mice		TCR γδ (UC7-13D5/GL4)			(57)
	C57BL/6 TCRδKO mice		TCR γδ (GL3), Vγ3 (536), Vγ4 (UC3-10A6)	Dermis	Produce IL-17, recruit neutrophils to the skin in the acute CHS reponse	(58)
	CHS model in CBA/J mice	DETC	TCR γδ (UC7-13D5), Vγ3 (536)	Lymph nodes and peritoneal cavity	Assist αβ T cells in the transfer of CHS in an allergen-unspecific manner	(55)
	Adoptive transfer of allergen-derivatized lymphocytes in CHS model in CBA/J mice		TCR γδ (GL3), Vγ3 (536)	Skin		(56)
	C57BL/6 TCRδKO mice and epidermis-derived DETC short-term cell line		TCR γδ (GL3), Vγ3 (536)	Epidermis	Produce IFN-γ and IL-17A following allergen treatment	(20)
	DETC cell line 7-17				Produce IFN-γ following allergen treatment	(59)
Human	Skin biopsies from allergen-induced skin lesions of healthy donors	Vδ2^+^ Vγ9^+^ T cells	TCR δ1, Vδ1 (δTCS1), Vδ2 (BB3), Vγ9 (TiyA)	Epidermis and dermis	Amplification or resolution of ACD	(60)
	Skin biopsies from patients with allergy to heavy metal salts				Mediate skin defense against highly reactive heavy metals	(61)
	Skin biopsies from patients with nickel allergy	γδ T cells	TCR γδ (γ3.20)		Produce IFN-γ, IL-17A and IL-22	(62)
**Anti-inflammatory role in ACD**						
Mouse	Allergen-derivatized Thy^+^ cells used for adoptive transfer to C57BL/6 mice	Thy1^+^ cells	Thy-1.2 (30-H12)	Epidermis	Ameliorate CHS response in an allergen-specific manner	(63)
	Allergen-derivatized/non-derivatized epidermis-derived cell lines AU4, AU16 used for adoptive transfer to C3H mice					(64)
	C57BL/6 TCRδKO mice and *in vivo* depletion with anti-TCRδ (UC7-13D5)	γδ T cells		Skin		(65)
	FVB TCRδKO mice	DETC	TCR γδ (GL3), Vγ3 (536)	Epidermis		(66)

The inflammatory role of γδ T cells in response to contact allergens was further underlined in studies using TCRδ-deficient mice (20, 58). Lack of γδ T cells resulted in more than a 50% reduction in the response to DNFB as measured by changes in ear-thickness (20, 58). Interestingly, the γδ T cells that assist the αβ T cells were shown to be DETC (55, 56). In accordance, we have shown that exposure of the skin to DNFB results in activation of DETC (20). The contact allergen-induced DETC activation was mediated by an indirect pathway, probably via the KC, involving IL-1β (20). In addition to DETC, the role of dermal γδ T cells in inflammatory skin disorders has been extensively investigated (58, 68–70). It has been shown that chimeric mice deficient in dermal γδ T cell but with normal amount of DETC have reduced ear swelling in response to DNFB treatment compared to WT mice. This indicated that dermal γδ T cells play an important pro-inflammatory role in the acute CHS response (58). In accordance, it was shown that dermal γδ T cells play a significant role in recruitment of neutrophils to the skin upon exposure to DNFB via an IL-17-dependent pathway (58).

Although these studies provide a strong evidence for a pro-inflammatory role of γδ T cells in the response to contact allergens, other studies have suggested an anti-inflammatory role of γδ T cells in ACD (63–66, 71). *Guan et al*. found that a lack of γδ T cells correlated with an increased ear swelling following OXA challenge (65). They used two different models to investigate this; TCRδ deficient C57BL/6 mice and depletion of the γδ T cells using the antibody UC7-13D5 (65). Interestingly, it has subsequently been shown that UC7-13D5 does not deplete γδ T cells, but instead induces TCR internalization and thereby generates “invisible” γδ T cells (72). Furthermore, as they only analyzed the response by changes in ear thickness, it is hard to conclude whether γδ T cells are in fact anti-inflammatory. In contrast, *Girardi et al*. observed no significant difference in ear thickness between TCRδ-deficient C57BL/6 mice and C57BL/6 mice following DNFB challenge (66). However, in the same study, an increase in ear thickness was found in TCRδ-deficient FVB-Tac mice compared to FVB-Tac mice using the same experimental setting. At first sight, this study suggested that DETC in C57BL/6 mice play a neutral role in ACD, whereas they play an anti-inflammatory role in FVB-Tac mice (66). However, it was subsequently shown that FVB-Tac mice have a defect in the development of DETC due to the lack of Skint-1 expression in the thymus (73–75). Interestingly, FVB-Tac mice develop spontaneous skin inflammation that correlates with infiltration of αβ T cells in the epidermis (73). Furthermore, an increased spontaneous skin inflammation was seen in TCRδ-deficient FVB-Tac mice compared to FVB-Tac mice (66). We have recently shown that a low-grade steady-state inflammation in mice with normal DETC increases the responsiveness to various contact allergens (76). Therefore, we find it difficult to conclude whether the increased response to contact allergens found in TCRδ-deficient FVB-Tac mice compared to FVB-Tac mice is directly mediated by an anti-inflammatory role of DETC or caused by the increased spontaneous skin inflammation observed in TCRδ-deficient FVB-Tac mice. *Sullivan et al*. found that intravenously (i.v.) injection of Thy-1^+^ epidermal cells primed with 2,4,6-trinitrobenzenesulfonic acid (TNBS) *in vitro* resulted in decreased ear swelling in C57BL/6 mice sensitized with TNCB, and that the size of the ear swelling was inversely correlated with the numbers of Thy-1^+^ epidermal cells injected (63). However, it was later shown that only 27% of Thy-1^+^ epidermal cells co-expressed Vγ3 (64). This makes it difficult to conclude whether the tolerance induction is mediated by DETC or by another cell type. In addition, it was shown that *in vitro* FITC-conjugated Thy-1^+^ epidermal cells clones (AU4 and AU16) could induce tolerance in FITC-challenged C3H mice when injected subcutaneously (s.c.) but not when injected i.v. (64). In contrast, unconjugated AU4 and AU16 were not able to suppress ear swelling when administered s.c. but only when injected i.v. However, whereas both AU4 and AU16 expressed Thy-1, neither of them expressed Vγ3 (64). Therefore, it is difficult to conclude that the induced tolerance is mediated by DETC. However, both studies showed that the tolerance was mediated in an allergen-specific manner (63, 64). Thus, it was found that downregulation of the CHS response occurred in C57BL/6 mice that received i.v. injections of TNBS-primed Thy-1^+^ epidermal cells prior to sensitization and challenge with TNBS but not in mice sensitized and challenged with OXA (63). Furthermore, FITC-conjugated AU16 cells ameliorated the ear swelling in C3H mice that were challenged with FITC, but not in the C3H mice challenged with DNFB (64).

The conflicting results on the role of γδ T cells in the response to contact allergens seen in studies using TCRδ-deficient mice, might as discussed above be due to the level of spontaneous inflammation seen depending on different genetic background (20, 58, 65, 66). However, the diverging results on the role of γδ T cells in the response to contact allergens were also seen in studies with TCRδ-deficient mice on a C57BL/6 background: a pro-inflammatory role (20, 58), an anti-inflammatory role (65) and no effect (66). The explanation for this might be the use of different mouse models to induce CHS. *Girardi et al*. and *Guan et al*. sensitized and challenged on different skin areas whereas *Nielsen et al*. and *Jiang et al*. sensitized and challenged on the same skin area. Finally, lack of γδ T cells have been shown to lead to a repopulation of the skin by other cells, including innate lymphoid cells (ILC) and different types of αβ T cells e.g., DETC-like αβ T cells within the epidermis (48, 77). Interestingly, dermal ILC3 and CD4^+^ T helper 17 cells (Th17) were found able to take over the functions of acutely depleted γδ T cells (77). Additionally, the repopulation is likely to depend on the microbiotic environment within the specific animal facility. Thus, further investigations using combination of various γδ T cells ablation strategies like conditional depletion of γδ T cells and TCRδ-deficient model, together with CHS model might bring new insights to the role of DETC during the response to contact allergens.

## Allergen-Induced Activation of DETC

DETC were first described as Thy-1^+^ dendritic epidermal T cells (37, 38) and since then their role has been intensively investigated in healthy skin (48, 52, 78) and in different skin pathologies (20, 59, 66, 67, 79–84). Interestingly, contact allergens cannot directly activate DETC but must act via an indirect pathway to activate the DETC (20). Contact allergen-induced stress molecules expressed or secreted by KC likely mediate the contact allergen-induced DETC activation.

With their dendrites, DETC monitor the microenvironment of the epidermis (Figure 2). Since each DETC is in direct contact to multiple KC, they respond rapidly to stressed and damaged KC (42, 59, 78, 85). In line with this, KC and epidermal cells (EC) primed with DNFB *in vitro* or *in vivo* can activate cultured DETC. Additionally, EC primed with different contact allergens like TNCB, OXA can activate DETC, whereas EC primed with irritants like sodium lauryl sulfate (SLS) and croton oil cannot activate DETC (67). If the DETC are physically separated from the EC by a cytokine permeable membrane, their proliferation rate is reduced (67), showing the importance of cell to cell contact for proper DETC activation. Several molecules, such as the NKG2D ligands mouse UL-16-binding protein-like transcript 1 (Mult-1), histocompatibility 60 (H60) and retinoic acid early inducible-1 (Rae-1) α-ε family in mice and MHC class I-chain-related A (MICA) in humans have been investigated for their ability to provide activation signals to DETC (59, 86–90). In the absence of cellular stress, the NKG2D ligands are scarcely expressed (91). However, following treatment with 2,4-dinitrobenzenesulfonic acid (DNBS) the murine KC cell line PAM2.12 upregulates Mult-1, H60 and Rae-1 (59). Likewise, treatment of mice with DNFB resulted in upregulation of Mult-1 in the epidermis (59). Importantly, DETC seem to be the only cells expressing NKG2D in the skin of mice (59). In humans, MICA is upregulated on primary KC following exposure to nickel (59). Interestingly, in addition to cutaneous lymphocyte-associated antigen (CLA) positive γδ T cells, a majority of CLA^+^ CD8^+^ T cells and of CLA^+^ natural killer (NK) cells express NKG2D in humans (59). Blocking NKG2D on DETC resulted in a reduction in allergen-induced IFN-γ production (59), which provides strong evidence for an important role of NKG2D and its ligands in allergen-induced DETC activation. In addition to NKG2D, two other co-stimulatory receptors, JAML and CD100, play important roles in DETC activation during wound repair (79, 87). Although, the role of JAML and CD100 have not been investigated in ACD, it may be speculated that they play a similar role in DETC activation and the inflammatory response in ACD as seen in wound repair.

## γδ T Cells in Human ACD

The role of γδ T cells in ACD in humans is poorly characterized. In normal adult skin, γδ T cells represent around 1–15% of the CD3^+^ lymphocytes (41, 60, 92, 93) with γδ T cells located within the basal KC layer of the epidermis and in the perivascular areas of the dermis. The localization of γδ T cells in perivascular areas suggests their origin from the circulation (39, 41, 92). In accordance, γδ T cells infiltrate the epidermis and dermis at later time point after challenge with DNCB, namely at 48 h post challenge (60). This suggested a role for γδ T cells in the amplification or the resolution of ACD reaction and not its initiation (60). In addition, γδ T cells are found in the epidermis and the dermis in allergic skin reactions to gold chloride and some of them exert dendritic morphology (61). Finally, challenge of the skin with nickel in nickel-allergic patients, resulted in a rise of γδ T cells that produced IFN-γ, IL-17A, and IL-22 in the dermis (62). This suggested a pro-inflammatory role of γδ T cells in the response to nickel. Although αβ T cells certainly are central mediators of ACD, more studies on human γδ T cells should be conducted to fully characterize their role in ACD.

## Targeting NKG2D as a Potential Novel Treatment for ACD

Avoidance of exposure to the specific contact allergen is the optimal preventive treatment and will ease the symptoms in many patients with ACD. However, some patients cannot avoid the contact allergen and others need active treatment in addition to allergen avoidance to resolve the symptoms. Thus, treatment with broad anti-inflammatory/immunosuppressive drugs such as corticosteroids and cyclosporine is crucial to restrain ACD symptoms. To date, no biologics have been proven successful in the treatment of ACD in man (94). A central role of NKG2D in responses to contact allergens suggests that targeting NKG2D might be a more specific way to treat ACD than the broad anti-inflammatory/immunosuppressive drugs used today (59). Thus, locally applied NKG2D blockers or antagonist should be tested for their use in treatment of ACD. Furthermore, it has been shown that ligation of NKG2D leads to DETC degranulation-mediated cytotoxicity (88) and consequently degranulation inhibitors might be alternative promising compounds in ACD. In brief, further basic and clinical studies investigating precision therapeutics are crucial for the development of more specific treatments of ACD.

## Conclusion

γδ T cells, including DETC, are crucial regulators of immune responses to contact allergens. Although some studies have suggested that γδ T cells might play an anti-inflammatory role during ACD, the majority of studies point to a pro-inflammatory role of γδ T cells in ACD. Contact allergens induce activation of DETC in mice via an indirect pathway that involves both cytokines and stress molecules expressed by KC which are subsequently recognized by NKG2D on the DETC. As human KC also produce and express cytokines and stress molecules in response to allergens, it is likely that these molecules could be potential targets in more specific treatments of ACD.

## Author Contributions

VM, CG and CB wrote the paper. VM made the table and figures.

## Conflict of Interest

The authors declare that the research was conducted in the absence of any commercial or financial relationships that could be construed as a potential conflict of interest.
